# Clinical assessment of the criticality index – dynamic, a machine learning prediction model of future care needs in pediatric inpatients

**DOI:** 10.1371/journal.pone.0320586

**Published:** 2025-04-30

**Authors:** Anita K. Patel, Taylor Olson, Christopher Ray, Eduardo A. Trujillo-Rivera, Hiroki Morizono, Murray M. Pollack

**Affiliations:** 1 Department of Pediatrics, Division of Critical Care Medicine, Children’s National Health System, Washington, District of Columbia, United States of America; 2 George Washington University School of Medicine and Health Sciences, Washington, District of Columbia, United States of America; 3 Department of Pediatrics, Division of Critical Care Medicine, Children’s Hospital of Richmond at the Virginia Commonwealth University, Richmond, Virginia, United States of America; 4 Children’s National Research Institute, Children’s National Hospital, Washington, District of Columbia, United States of America; 5 Department of Genomics and Precision Medicine, George Washington University School of Medicine and Health Sciences, Washington, District of Columbia, United States of America; Université Paris 13 UFR de Santé Médecine Biologie Humaine: Universite Sorbonne Paris Nord UFR de Sante Medecine Biologie Humaine, France

## Abstract

**Objective:**

To assess patient characteristics and care factors that are associated with correct and incorrect predictions of future care locations (ICU vs. non-ICU) by the Criticality Index-Dynamic (CI-D), with the goal of enhancing the CI-D.

**Design:**

Retrospective structured chart review

**Participants:**

All pediatric inpatients admitted from January` 1^st^ 2018 – February 29^th^ 2020

through the emergency department.

**Main outcome(s) and measure(s):**

Patient characteristics and care factors associated with correct (true positives, true negatives) and incorrect predictions (false positives, false negatives) of future care locations (ICU vs. non-ICU) by the CI-D were assessed.

**Results:**

Of the 3,018, patients, 139 transitioned from non-ICU locations to ICU care; 482 were transferred from the ICU to non-ICU care locations, and 2,400 remained in non-ICU care locations. For the ICU Prediction group, the false negative patients were older, more frequently male, and had longer hospital and ICU lengths of stay compared to the true positive patients. The significant differences in the ICU Prediction group for false negative compared to the true positive patients included a less frequent: primary diagnosis of respiratory failure, use of high flow nasal canula, hourly cardio-respiratory vital signs prior to transfer to the ICU, and neurologic vital signs after transfer from the ICU. For the ICU Discharge prediction group, false positive patients were more frequently: younger, had a primary diagnosis of respiratory failure, more frequently received respiratory support after discharge from the ICU, and received less frequent neurological vital signs prior to transfer from the ICU. For the Non-transfer prediction category, demographics and clinical variables did not differ between the true negative and false positive prediction groups.

**Conclusion and relevance:**

We conducted the first comprehensive analysis via structured chart reviews of patient characteristics and care factors that are associated with correct and incorrect predictions of future care locations by the machine learning algorithm, the CI-D, gaining insights into potential new predictor variables for inclusion in the model to improve future model iterations.

## Introduction

Rigorous assessment including clinical validation of machine learning algorithms is a necessary step in their development and deployment in healthcare settings that will help ensure the algorithms are accurate, reliable, and safe for clinical use [1–4]. Many machine learning models have demonstrated overall excellent performance in a variety of diseases and conditions, usually related to predicting the presence of a condition and/or its prognosis [5–8].

However, machine learning algorithms have downsides that may inhibit their clinical utility, especially their application to individual patients [9]. First, they are generally opaque [10]. Unlike traditional, statistically derived algorithms, machine learning algorithms are difficult to explain or justify. Second, the algorithms are highly contextualized; that is, they are fitted to specific situations. This specificity may decrease performance when the algorithms are applied to new environments. For example, data elements adapted from national or international datasets when applied to single institutions may have differences that impose error and limit applicability. This difficulty is especially relevant to algorithms assessing operational or other activity that may be institutionally dependent [11,12]. Third, commonly used performance metrics such as area under the receiver operating characteristic curve (AUROC) are most relevant to performance in populations, not individual patients.

Trust in machine learning algorithms may be enhanced by assessing their clinical performance at the individual patient level prior to deployment. Performance assessment at the patient level may detect patient groups where performance is suboptimal. Recently, we developed a machine learning method, the Criticality Index – Dynamic (CI-D), to assess the potential for pediatric inpatient deterioration by assessing the risk for needing intensive care up to 30 hours in the future [12,13].

The current study extends the traditional machine learning validation phase to include a patient-level assessment prior to deployment in three clinically relevant prediction groups, transition from: non-ICU ◊ICU care, ICU care ◊ non-ICU care, non-ICU care ◊ non-ICU care. The primary aim was to assess patient characteristics and care factors that are associated with correct (true positives, true negatives) and incorrect predictions (false positives, false negatives) of potential transition to future care locations (ICU vs. non-ICU) in three clinically relevant prediction groups by the CI-D, with the goal of enhancing the CI-D.

## Methods

### Setting

The study was conducted at Children’s National Hospital, a pediatric hospital with 323 beds inclusive of 48 bed Pediatric ICU beds and 24 Cardiac ICU beds. The patient sample included all inpatient admissions directed or coordinated through the Emergency Department to the inpatient service from January 1, 2018 to February 29, 2020 and corresponded to the test sample for the CI-D validation in this institution [12]. Patients admitted through the Emergency Department were selected because there is often uncertainty in predicting their clinical needs. Exclusions included patients over 21 years of age and those admitted to the neonatal ICU. All chart reviews were performed from October 1^st^, 2022 to April 1^st^, 2023. This study was approved by the Institutional Review Board at Children’s National Hospital (protocol# Pro00015931).

### Prediction model (Criticality Index – Dynamic (CI-D))

The Criticality Index – Dynamic is a neural network machine learning model using physiology (vital signs and laboratory variables), therapies (medications and mechanical ventilation), and intensity of care (reflected by number and frequency of medications, labs and vital signs) [12,14]. The model predicts future care location as ICU or non-ICU in the future >6–12 hours, >12–18 hours, >18–24 hours, and > 24–30 hours using separate algorithms. The Criticality Index – Dynamic, was initially developed and tested in a national sample of over 120,000 patients and then optimized for this single site [12]. Performance metrics in the single site test sample for care location in the >6–12 hour future time prediction were an area under the receiver operating characteristic (AUROC) curve = 0.944, area under the precision recall (AUPR) curve = 0.791, and, at a 90% sensitivity, accuracy = 85.7%, specificity = 0.846, precision = 0.603, and number needed to evaluate = 1.66 [12]. The 90% sensitivity was chosen because it reflects a clinically reasonable balance of true positive and false negative future care location predictions while maintaining a low number needed to evaluate (1/precision = 1.66).This chart review study aimed at assessing patient characteristics and care factors associated with correct and incorrect predictions of ICU and non-ICU care locations in 3 prediction groups for the future >6–12 hour time period, the time period with the most relevance to early warning systems and clinical care [15].

### Chart review methodology

We assessed the patient characteristics and care factors using chart reviews of correct and incorrect predictions of the CI-D in the three clinically relevant prediction groups (transition to: non-ICU ◊ICU care, ICU care ◊ non-ICU care, non-ICU care ◊ ICU care). Table 1 details the prediction groups, and the correct (true) and incorrect (false) prediction outcome categories for each group. The ICU care location was considered a positive prediction and the non-ICU care locations was a negative prediction. The three prediction groups assessed were: ICU Admissions (admission from non-ICU to ICU care); ICU Discharges (transfer from ICU to non-ICU care); and Non-transfers (care in a non-ICU location without transfer to the ICU). We did not assess patients admitted directly to the ICU. We chose the prediction outcome groups compared based on the “true” future location of the patient. For ICU Admissions, the outcome categories were true positives (patients correctly predicted to transition from non-ICU to ICU care) and false negatives (patients incorrectly not predicted to transition from non-ICU to ICU care). For ICU Discharges, the outcome categories were true negatives (patients correctly predicted to transition from ICU to non-ICU care) and false positives (patients incorrectly not predicted to transition from ICU to non-ICU care). For Non-transfers, those cared for in routine care, the outcome categories were true negatives (patients correctly predicted to remain in a non-ICU care units) and false positives (patients incorrectly predicted to transition to the ICU care but who did not transition). Chart reviews were performed for all patients in the ICU Admission prediction group; all other patients reviewed were randomly selected (see below) from their prediction outcome group and prediction outcome category.

**Table 1:** Prediction groups and prediction outcome categories. The three prediction groups were transfer from non-ICU to ICU care, transfer from ICU to non-ICU care, and non-ICU to non-ICU care (no transfer). The table lists the correct and incorrect prediction outcome categories for each prediction group and their sample size (see text for detailed explanation).

		Prediction Outcome Categories			
True Transition Status	Prediction Group (n = 339)	True Positive: correct predictions for ICU care (n = 104)	False Positive: incorrect predictions for ICU care (n = 100)	False Negative: Incorrect predictions for non-ICU care (n = 35)	True Negative: Correct predictions for non-ICU care (n = 100)
**Non-ICU care to ICU care**	ICU Admission (n = 139)	correct prediction of transfer to ICU (n = 104) (1)	n/a	incorrect prediction of transfer to non-ICU location when true transition is to ICU (n = 35) (1)	n/a
**ICU to non-ICU**	ICU Discharge (n = 100)	n/a	incorrect prediction of remaining in ICU care when true transition is to non-ICU care (n = 50) (2)		correct prediction of transfer to non-ICU (n = 50) (2)
**Non-ICU, never transferred**	Non-Transfer (n = 100)	n/a	incorrect prediction of transfer to ICU (n = 50) (3)	n/a	correct prediction of non-transfer (n = 50) (3)

True = correct prediction; False = incorrect prediction; Positive = ICU care area; Negative = non-ICU care area

(1) = ICU Admission Questionnaire; (2) = ICU Discharge Questionnaire; (3) = Non-transfer Questionnaire

The structured chart review methodology was based on that proposed and tested by Siems et al. [16] Structured chart reviews detail the areas of the medical record to be reviewed and recommendations for the time requirements [17]. All reviews were completed by two reviewers (TO, CR) who had greater than one year of pediatric critical care training at time of the chart review. The structured chart review responses were recorded in a REDCap database. An instructional session was held by the principal investigator (AKP) with both reviewers, followed by direct supervision of the initial five reviews. Each review form provided instructions detailing the location(s) within the electronic health record (EHR) to assess each question. EHR locations included the admission and discharge notes, procedure notes, diagnosis and problem lists, vital sign charting, respiratory orders and charting, and the medication administration record. The recommended time to complete each review was 10–15 minutes. Interrater reliability was assessed by Cohen’s kappa-statistic for those questionnaire items with quantitative responses with a pre-determined minimum acceptable kappa statistic of 0.61.

The number of chart reviews for the prediction groups was based on Johnston et al.’s assessment for sample sizes in descriptive retrospective studies targeting subgroups of interest [18]. A total of 339 patients (Table 1) were evaluated including the entire sample of the ICU Admission group (n = 139) and 100 reviews in each of the two remaining prediction groups (50 in each of the two outcome categories for each prediction group). The total sample of the ICU Admission prediction group was evaluated as this is the most clinically relevant group representing the model’s potential as an early warning method.

The purpose of the chart reviews was to assess patient characteristics and care factors associated with correct or incorrect predictions of future care location. The reviews were conducted using separate questionnaires for each prediction group: the ICU admission group assessed the 24 hours prior to and 24 hours after ICU admission, the ICU discharge group assessed the 24 hours prior to and 24 hours after ICU discharge, and the non-transfer (non-ICU care) group assessed a single, randomly selected 24-hour time period for randomly selected patients. The questionnaires had both identical elements and unique elements relevant to the prediction group (Table 2). Questionnaire items included those associated with nursing, respiratory care, and physician-led care. The assessments categories are shown in Table 2. Each questionnaire included a qualitative assessment regarding the reason(s) for care location placement that was categorized by organ system. The ICU Admission questionnaire included an assessment of why the patient required admission to the ICU with prompts requesting the reviewer to assess issues related including the frequency and/or intensity of care items. The ICU Discharge questionnaire included an assessment of why the patient was discharged from the ICU, with prompts requesting the reviewer to assess issues related to nursing, respiratory care, and resolution of ICU-specific therapies and procedures relative to the transfer including the frequency and/or intensity of care items. The ICU Discharge questionnaire also tabulated the need for re-admission to the ICU within 24 hours of discharge. The Non-transfer (non-ICU care only) questionnaire included an assessment of frequency and intensity of nursing care, respiratory care and physician care, and if these modalities were of appropriate frequency or intensity for non-ICU care. All three questionnaires had a free text area for comments. The time required to complete the individual patient review was also recorded. The kappa statistics for the ICU Admission, ICU Discharge and Non-transfer questionnaires were 0.86, 0.91, and 0.83, respectively.

**Table 2:** Care factors associated with each prediction group.

	Prediction Group		
Questionnaire Items	ICU Admission	ICU Discharge	Non-transfer^1^
Primary diagnosis^2^	x	x	x
Secondary diagnoses^2^	x	x	x
Tertiary diagnoses^2^	x	x	x
Vital sign frequency prior to and after transfer	x	x	x
Neurologic assessment frequency prior to and after transfer	x	x	x
Highest respiratory support prior to and after transfer^3^	x	x	x
Tracheostomy and support^4^	x	x	x
Medications categories prior to and after transfer^5^	x	x	x
ICU specific medications^6^	x	x	
Procedures^7^	x	x	
Cardiac arrest prior to transfer	x	x	
ICU readmission		x	
Qualitative assessment of reason for ICU admission/discharge	x	x	
Qualitative reviewer assessment	x	x	x

^1^Time periods for non-transfers were randomly selected 24-hour time periods and the following 24-hour time period.

^2^Primary, secondary and tertiary diagnoses underlying reason for ICU or hospital admission were determined based on clinical judgement after reviewing the admission and discharge notes and associated problem lists.

^3^Respiratory support options included: nasal cannula <4L, high flow nasal cannula, continuous positive airway pressure, bi-level positive airway pressure, and mechanical ventilation.

^4^If a patient had a tracheostomy, options for level of support included: none, humidified air, oxygen, continuous positive airway pressure, bi-level positive airway pressure, or mechanical ventilation.

^5^Medication categories assessed included: inhaled beta agonists, insulin, opioids, anti-seizure medications, anti-secretion medications, diuretics, anti-hypertensive medications, and cardiac medications.

^6^ICU specific medications included: inotropes and/or vasopressors, continuous albuterol, anti-hypertensive medications, sedation and/or analgesic medications, and endocrine/metabolic infusions.

^7^Procedures included: central line, arterial line, dialysis, pericardiocentesis, extra corporeal membrane oxygenation, intracranial pressure monitor and/or external ventricular drain.

### Statistical analyses

Demographic and questionnaire data were summarized using descriptive statistics. Continuous variables are expressed as medians with interquartile ranges, and categorical variables are expressed with counts and percentages. The dependent variables assessed were true positive, true negative, false positive and false negative CI-D predictions. The care factors associated with the dependent variables were compared within the selected clinically relevant prediction groups based on the patient’s true future location: non-ICU→ICU transfer (true positives vs. false negatives, i.e., impacting sensitivity), ICU→non-ICU transfer (true negatives vs. false positives, i.e., impacting specificity of ICU discharge), non-ICU→non-ICU non-transfers (true negatives vs. false positives, i.e., impacting specificity of non-transfer). The Mann Whitney U test was used to compare continuous measures and the Barnard CSM exact test [19] was used to compare categorical measures and proportions in Table 3 and S1 and S2 Appendices within the aforementioned prediction groups. The multinomial exact test was used to compare proportions when a patient could be represented by multiple categories within a division of care factors in S2 Appendix (i.e., a patient could have received medications in multiple subcategories). The Bonferroni correction was applied to control for type 1 error within each care factor division in Table 3 and S2 Appendix. Cohen’s kappa was computed to measure inter-rater reliability. All statistical analyses were performed in R studio [20].

**Table 3:** Demographic and questionnaire data for the ICU admission and ICU discharge prediction groups for the true and false outcome prediction categories. Only questionnaire data in sections with statistical or clinical significant differences are shown.

	Prediction Outcome Groups					
	ICU ADMISSION^1^			ICU DISCHARGE^1^		
Demographic Variables and Care Factor Divisions	True Positive: correct predictions for transfer to the ICU	False Negative: incorrect predictions for no transfer to the ICU	p-value^2^	True Negative: correct predictions for transfer out of the ICU	False Positive: incorrect predictions for no transfer out of the ICU	p-value^2^
**n**	104	35		50	50	
**Demographics**						
**Age (months)** ^3^	19.5 (6.8–121.5)	114 (10.0–201.5)	**0.02**	70.5 (20.5–166.5)	17.5 (7.0–89.0)	0.01
**Female (n (%))**	43 (41.3)	9 (25.7)	NS	22 (44.0)	21 (42.0)	0.92
**Black**	51 (49.0)	11 (31.4)	NS	20 (40.0)	14 (28.0)	0.27
**White**	8 (7.7)	5 (14.3)	NS	11 (22.0)	13 (26.0)	0.76
**Other-Unknown**	45 (43.3)	19 (54.3)	NS	19 (38.0)	23 (46.0)	0.48
**Hospital LOS (days)** ^**2**^	6.3 (2.6–18.7)	10.7 (4.5–14.8)	**0.01**	3.6 (2.3–7.7)	2.9 (2.1–5.9)	0.38
**ICU LOS (days)** ^**2**^	2.9 (1.4–13.8)	3.5 (1.5–13.8)	NS	1.5 (0.8–2.8)	1.5 (0.9–2.3)	0.80
**Diagnoses**						
**Primary Diagnosis: Respiratory Failure**	65 (62.5)	14 (40.0)	NS	19 (38.0)	36 (72.0)	<0.01
**Primary Diagnosis:** **Neurologic Disorders**	16 (15.4)	9 (25.7)	NS	7 (14.0)	3 (6.0)	NS
**Secondary Diagnosis:** **Non-Septic Infections**	26 (25.0)	11 (31.4)	NS	7 (14.0)	7 (14.0)	NS
**Cardio-Respiratory Vital Sign Assessment**						
**Prior to Transfer: hourly**	67 (64.4)	13 (37.1)	<0.01	26 (52.0)	31 (62.0)	NS
**Neurologic Vital Sign Assessments**						
**Prior to Transfer: hourly**	15 (14.4)	10 (28.6)	NS	14 (28.0)	3 (1.9)	0.04
**Prior to Transfer: every 4 hours**	26 (25.0)	3 (14.3)	NS	19 (38.0)	33 (66.0)	<0.01
**Respiratory Support**						
**Prior to Transfer: high flow nasal canula**	30 (28.8)	1 (2.9)	<0.01	14 (28.0)	23 (46.0)	NS
**After Transfer: NC oxygen <4 L/min**	17 (16.3)	9 (25.7)	NS	16 (32.0)	33 (66.0)	<0.01
**Medication Categories**						
**Prior to Transfer: inhaled beta agonists**	52 (50.0)	7 (20.0)	NS	0 (0.0)	1 (2.0)	NS
**Prior to Transfer:** **Opioids**	13 (12.5)	11 (31.4)		10 (20.0)	8 (16.0)	
**After Transfer:** **Pressors/Inotropes**	10 (9.6)	0 (0.0)	NS	0 (0.0)	1 (2.0)	NS

^1^There were no differences between the true and false groups for the ICU Admission and ICU Discharge prediction groups for the following categories: Tracheostomy, Tracheostomy Respiratory support, Medications, Procedures, or Cardiac Arrest.

^2^Univariate comparisons were completed using the Barnard’s exact test. The Bonferroni correction was used to adjust p-values within each care factor division to control for Type 1 error.

^3^Median (interquartile range)

## Results

Of the 3,018, patients, 139 transitioned from non-ICU locations to ICU care; 482 were transferred from the ICU to non-ICU care locations, and 2,400 remained in non-ICU care locations. The demographic description of this population has been previously published [12].

Table 3 lists demographic data and clinical care factors for the ICU Admission and ICU Discharge prediction groups within care factor divisions where there is a statistical or clinical difference between the true and false outcome prediction categories. Complete data for all three prediction groups are given in S1 Appendix (demographic data by prediction group) and S2 Appendix (questionnaire care factor data). For the ICU Prediction group, the false negative patients were older, more frequently male, and had longer hospital and ICU lengths of stay compared to the true positive patients. While most clinical care factors were similar, the differences in the ICU Prediction group for the false negative patients compared to the true positive patients included a less frequent: primary diagnosis of respiratory failure, use of high flow nasal canula prior to transfer to the ICU, hourly cardi-respiratory vital signs prior to transfer to the ICU, administration of inhaled beta agonists prior to transfer to ICU, and administration of pressors and/or inotropes after transfer to ICU. False negative patients compared to true positive patients also experienced more frequent: primary diagnosis of respiratory failure, secondary diagnosis of non-septic infections, hourly neurologic vital signs prior to transfer to ICU, and administration of opioids prior to transfer to ICU. For the ICU Discharge prediction group, false positive patients were more frequently: younger, more frequently had a primary diagnosis of respiratory failure, more frequently received respiratory support after discharge from the ICU, and received less frequent neurological vital signs prior to transfer from the ICU.

For the Non-transfer prediction category, demographics and clinical variables did not differ between the true negative and false positive prediction groups (S1 and S2 Appendices).

A qualitative assessment of reasons for transfer or non-transfer sorted by prediction group and organ system is shown in S3 Appendix. Selected reasons with greater than one occurrence for transfer or non-transfer are detailed here (all reasons are detailed in S3 Appendix). For the ICU Admission prediction group, 23/35 false negative predictions occurred most frequently in the respiratory and neurologic care groups. Care factors with differences between true positive and false negative predictions in the respiratory and neurologic care groups were absence of acute and/or chronic mechanical ventilator support (n = 2), and concern for increased intracranial pressure (ICP) post operative craniotomy care (n = 4). In the other organ support groups, issues included arrythmia management (n = 3), and care of a ruptured abdominal organ (n = 2). For the ICU Discharge prediction group, there were no consistent differences in reason for transfer between the true negative and false positive predictions. The single issue in both true negative and false positive predictions was resolution of the patient’s reason for ICU care. For the Non-transfer group, 22/50 false positive predictions were in the domain of respiratory support, including the issues of prior use of high flow nasal cannula in the ICU earlier during the hospitalization (n= 2) and continuous albuterol use in patients greater than 3 years old (n= 3).

## Discussion

We conducted a comprehensive analysis via structured chart reviews of patient characteristics and care factors that are associated with correct and incorrect predictions of future care locations (ICU vs. non-ICU) by the Criticality Index – Dynamic with the goal of finding patient groups whose predictions might be improved in future iterations of the machine learning model. For the ICU Admission prediction group, false negative patient demographics were characterized by higher age, higher male prevalence, and longer hospital and ICU stays compared to their true positive counterparts. Notably, differences were observed in primary diagnosis, use of high-flow nasal cannula, frequency of cardio-respiratory vital sign monitoring, post-ICU neurological vital sign assessments, and administration certain classes of medications. In the ICU Discharge prediction group, false positive patients were predominantly younger, had a higher incidence of respiratory failure as a primary diagnosis, relied more on post-ICU respiratory support, and underwent less frequent neurological vital sign monitoring prior to ICU discharge. The non-transfer group exhibited only a significant age difference between true negative and false positive patients, with the latter being younger. These findings enhance our understanding of the factors influencing prediction outcomes and offer valuable guidance for the refinement and customization of the Criticality Index – Dynamic algorithm to optimize its predictive capabilities for specific patient subgroups.

The contextualized nature of machine learning algorithms underscores a fundamental characteristic that distinguishes them from traditional statistical models. These algorithms are frequently trained and fine-tuned to specific situations or datasets, capturing patterns and correlations within that context to make predictions or decisions. Data elements drawn from national or international datasets, for instance, may carry nuances or contextual differences that do not align with the specifics of individual institutions or clinical practices. The CI-D was initially developed and validated using a national dataset, and the original architecture inclusive of identical variables was re-trained and calibrated using this single-institution data to improve the model’s ability to detect institutional patterns. Although the institutional model performed well (AUROC = 0.994, AUPR = 0.791), the chart review highlighted that certain institutional care patterns may not have been adequately captured. For example, this institution allows continuous albuterol to be administered in inpatient care areas if patients are over the age of 3, while other institutions frequently require continuous albuterol to be administered in ICU care areas. Despite the model recalibration, this care pattern was not detected. Future iterations of the model will include enhanced respiratory support items (i.e., nasal cannula, face mask, high flow nasal cannula, etc) to capture patterns of care that are unique to this single institution. Additionally, enhanced attention to monitoring neurologic status is indicated based on the ICU Admission and ICU Discharge prediction groups.

Machine learning algorithms are frequently opaque, which can present unique challenges in their application to clinical settings. The CI-D model architecture is a neural network, a method frequently referred to as a ‘black box’ because it is difficult to provide clear explanations or justifications for the model’s predictor variables. This opacity can pose significant hurdles in healthcare, where interpretability and transparency are important for healthcare professionals to trust and act upon algorithmic assessments. The structured chart review revealed some potential improvements for patients with respiratory and neurologic disease pathologies that can be addressed with additional model inputs, differential weighting of certain model features, and close assessment of variable importance in the model. Most important, the model generally captures patient care factors and disease characteristics that necessitate both ICU care and ICU discharge across a wide spectrum of disease pathologies with minimal systemic deficiencies. As healthcare increasingly embraces machine learning, defining a clearly delineated path to integration of these algorithms into patient care is important to ensure the safe, effective, and ethical integration of these algorithms into patient care.

Enhancing trust in machine learning algorithms within healthcare may hinge on a critical step: assessing their clinical performance at the individual patient level prior to deployment. This study’s approach represents a focus on individualized patients rather than patient populations. By subjecting algorithms to scrutiny at the patient level, healthcare providers gain valuable insights into the algorithm’s strengths and weaknesses. This granular assessment fosters a deeper understanding the algorithm’s performance and highlights patient groups where its performance could be improved. Traditional performance metrics, such as AUROC and AUPR are population-based statistics that do not assess model performance in individual patients and disease groups. Individual patient assessment could be an important step in ensuring that machine learning algorithms are accurate and reliable and acceptable to healthcare providers.

## Limitations

There are several limitations to this clinical assessment of the CI-D. Firstly, the subjective assessment relied on the judgement of 2 individual reviewers with high response concordance, other practitioners may have had different perceptions. Secondly, while our chart review methodology was developed to identify care, treatment, and diagnostic factors associated with correct and incorrect predictions, it is possible that there are other factors we did not assess. Others using this approach should assess the appropriateness of the items we selected for their sites. Third, we have not yet developed a new model with variables representing the potential improvements found in this analysis. Fourth, the results of this chart review may not be applicable to other hospital systems as criteria for admission and discharge to and from the ICU vary considerably among institutions.

## Conclusion

We conducted the first comprehensive analysis via structured chart reviews of patient characteristics and care factors that are associated with correct and incorrect predictions of future care locations by the machine learning algorithm, the Criticality Index – Dynamic [12], gaining insights into potential new predictor variables for inclusion in the model to improve future model iterations. Importantly, this chart review highlighted that there were few systematic reasons for incorrect model predictions, suggesting that it is appropriate for clinical application. This chart review methodology is a valuable framework for assessing machine learning models in individual patients that can be applied to machine learning models in other clinical environments.

## Supporting information

S1 Appendix Demographic Characteristics of Patients in the ICU Admission, Discharge and Non-Transfer Prediction Groups. “True” is the correct prediction and “false” is the incorrect prediction. “Positive” is the ICU and “negative” is a non-ICU care area.
(DOCX)

S2 Appendix Care Factor Results from the Structured Chart Review for ICU Admission, Discharge and Non-Transfer Prediction Groups. Data from sections with statistical and/or clinical significance are also shown in Table 3.
(DOCX)

S3 Appendix Reasons for ICU Admission, Discharge, or Non-ICU care. “True” is the correct prediction and “false” is the incorrect prediction. “Positive” is the ICU care location and “negative” is a non-ICU care location.
(DOCX)
