# Supplementary material for: Clinical assessment of the criticality index – dynamic, a machine learning prediction model of future care needs in pediatric inpatients
Source: PLoS One. 2025 Apr 30;20(4):e0320586. doi: 10.1371/journal.pone.0320586 (PMC12043114; doi:10.1371/journal.pone.0320586)
Supplement: S1 Appendix — “True” is the correct prediction and “false” is the incorrect prediction. “Positive” is the ICU and “negative” is a non-ICU care area. (DOCX) [file pone.0320586.s001.docx]

Appendix 1: Demographic Characteristics of Patients in the ICU Admission, Discharge and Non-Transfer Prediction Groups. “True” is the correct prediction and “false” is the incorrect prediction. “Positive” is the ICU and “negative” is a non-ICU care area.

| **Characteristic** | **All** | **Chart Review Patients** | **p-value** | **ICU Admission** | **ICU Admission** | **p-value** | **ICU Dispo** | **ICU Dispo** | **p-value** | **Inpatient** | **Inpatient** | **p-value** |
| --- | --- | --- | --- | --- | --- | --- | --- | --- | --- | --- | --- | --- |
|  |  |  |  | **True Positive** | **False Negative** |  | **True Negative** | **False Positive** |  | **True Negative** | **False Positive** |  |
| Patients | 3018 | 339 |  | 104 | 35 |  | 50 | 50 |  | 50 | 50 |  |
| Age (Months)a | 71 (16 - 159) | 29 (7.0-123.0) | <0.01 | 19.5 (6.8-121.5) | 114 (10.0-201.5) | 0.02 | 70.5 (20.5-166.5) | 17.5 (7.0-89.0) | <0.01 | 36.0 (3.3-86.5) | 17.0 (3.0-79.5) | 0.44 |
| Female (n (%)) | 1470 (47.2) | 131 (38.6) | 0.06 | 43 (41.3) | 9 (25.7) | 0.25 | 22 (44.0) | 21 (42.0) | 0.92 | 19 (38.0) | 17 (34.0) | 0.76 |
| Black | 1444 (46.3) | 137 (40.4) | <0.05 | 51 (49.0) | 11 (31.4) | 0.17 | 20 (40.0) | 14 (28.0) | 0.27 | 21 (42.0) | 20 (40.0) | 0.92 |
| White | 637 (20.4) | 59 (17.4) | 0.72 | 8 (7.7) | 5 (14.3) | 0.34 | 11 (22.0) | 13 (26.0) | 0.76 | 11 (22.0) | 11 (22.0) | 1 |
| Other-Unknown | 933 (31.0) | 143 (42.2) | 0.09 | 45 (43.3) | 19 (54.3) | 0.68 | 19 (38.0) | 23 (46.0) | 0.48 | 18 (36.0) | 19 (38.0) | 0.92 |
| Hospital LOS (days)a | 2.4 (1.2-4.6) | 4.1 (2.3-9.7) | <0.01 | 6.3 (2.6-18.7) | 10.7 (4.5-14.8) | <0.01 | 3.6 (2.3-7.7) | 2.9 (2.1-5.9) | 0.38 | 3.1 (1.9-5.9) | 3.2 (1.9-6.0) | 0.71 |
| ICU LOS (days)a | 1.9 (1.0-3.6) | 1.9 (1.0-3.6) | <0.01 | 2.9 (1.4-13.8) | 3.5 (1.5-13.8) | 0.84 | 1.5 (0.8-2.8) | 1.6 (0.9-2.3) | 0.80 | N/A | N/A |  |
| a. Median (interquartile range) | | | | | | | | | | | |  |
