# Supplementary material for: Clinical assessment of the criticality index – dynamic, a machine learning prediction model of future care needs in pediatric inpatients
Source: PLoS One. 2025 Apr 30;20(4):e0320586. doi: 10.1371/journal.pone.0320586 (PMC12043114; doi:10.1371/journal.pone.0320586)
Supplement: S2 Appendix — Data from sections with statistical and/or clinical significance are also shown in Table 3. (DOCX) [file pone.0320586.s002.docx]

Appendix 2. Care Factor Results from the Structured Chart Review for ICU Admission, Discharge and Non-Transfer Prediction Groups. Data from sections with statistical and/or clinical significance are also shown in Table 3.

|  | **Prediction Outcome Groups** | | |
| --- | --- | --- | --- |
| **Transfer Group: ICU ADMISSION**  **Care Factor Divisions** | **True Positive: Correct Predictions for transfers to the ICU (n = 104)** | **False Negative: Incorrectly predicted not to transfer to the ICU (n = 35)** | **adjusted p-value (1)** |
| Primary Diagnosis | | | |
| Respiratory Failure | 65 (62.5) | 14 (40.0) | **NS** |
| Neurologic Disorders | 16 (15.4) | 9 (25.7) | **NS** |
| Cardiac Disorders | 10 (9.6) | 4 (11.4) | **NS** |
| Post-Operative Care | 8 (7.7) | 1 (2.9) | **NS** |
| Septic Shock | 4 (3.8) | 1 (2.9) | **NS** |
| Non-Septic Infection | 1 (1.0) | 3 (8.6) | **NS** |
| Trauma | 0 (0.0) | 1 (2.9) | **NS** |
| Secondary Diagnosis | | | |
| Non-Septic Infection | 26 (25.0) | 11 (31.4) | NS |
| Cardiac | 10 (9.6) | 0 (0.0) | NS |
| Respiratory Failure | 5 (4.8) | 1 (2.9) | NS |
| Post-Operative Care | 3 (2.9) | 0 (0.0) | NS |
| Neurologic Disorders | 2 (1.9) | 3 (8.6) | NS |
| Gastro-Intestinal Disorders | 2 (1.9) | 3 (8.6) | NS |
| Septic Shock | 2 (1.9) | 0 (0.0) | NS |
| Trauma | 1 (1.0) | 0 (0.0) | NS |
| Oncology | 0 (0.0) | 3 (8.6) | NS |
| Tertiary Diagnosis | | | |
| Respiratory Failure | 7 (6.7) | 1 (2.9) | NS |
| Oncology | 2 (1.9) | 0 (0.0) | NS |
| Post-Operative Care | 2 (1.9) | 0 (0.0) | NS |
| Non-Septic Infection | 2 (1.9) | 1 (2.9) | NS |
| Metabolic Disorder | 1 (1.0) | 0 (0.0) | NS |
| Gastro-Intestinal Disorders | 1 (1.0) | 0 (0.0) | NS |
| Hematology | 0 (0.0) | 1 (2.9) | NS |
| Frequency of Vital Signs 24 Hours Prior to Transfer to ICU | | | |
| qh1 | 67 (64.4) | 13 (37.1) | **0.03** |
| q2h | 11 (10.6) | 3 (8.6) | NS |
| q3h | 2 (1.9) | 3 (8.6) | NS 0.5 |
| q4h | 4 (3.8) | 2 (5.7) | NS 0.85 |
| q6h | 1 (1.0) | 0 (0.0) | NS |
| q8h | 1 (1.8) | 0 (0.0) | NS |
| Frequency of Vital Signs in the First 24 Hours after Transfer to the ICU | | | |
| q1h | 96 (94.2) | 31 (88.6) | NS |
| q2h | 1 (1.0) | 0 (0.0) | NS |
| q4h | 1 (1.0) | 3 (8.6) | NS |
| Frequency of Neurologic Assessments 24 Hours Prior to Transfer to the ICU | | | |
| q1h | 3 (4.8) | 3 (14.3) | NS |
| q2h | 11 (10.6) | 6 (17.1) | NS |
| q3h | 5 (4.8) | 5 (14.3) | NS |
| q4h | 26 (25.0) | 3 (14.3) | NS |
| q6h | 3 (2.9) | 0 (0.0) | NS |
| q8h | 1 (1.0) | 2 (5.7) | NS |
| q12h | 17 (16.3) | 10 (2.9) | NS |
| q24h | 12 (11.5) | 2 (5.7) | NS |
| Frequency of Neurologic Assessments 24 Hours Post Transfer to the ICU | | | |
| q1h | 15 (14.4) | 10 (28.6) | NS |
| q2h | 17 (16.3) | 7 (20.0) | NS |
| q3h | 7 (6.7) | 2 (5.7) | NS |
| q4h | 60 (57.7) | 13 (37.1) | NS |
| q6h | 1 (1.0) | 0 (0.0) | NS |
| q12h | 2 (1.9) | 2 (5.7) | NS |
| Highest Level of Respiratory Support 24 Hours Prior to Transfer to the ICU | | | |
| NC Oxygen < 4L/min | 17 (16.3) | 9 (25.7) | NS |
| NC Oxygen > 4L/min | 4 (3.8) | 0 (0.0) | NS |
| HFNC | 30 (28.8) | 1 (2.9) | **<0.01** |
| CPAP | 1 (1.0) | 0 (0.0) | NS |
| BIPAP | 10 (9.6) | 0 (0.0) | NS |
| Mechanical Ventilation | 8 (7.7) | 0 (0.0) | NS |
| Highest Level of Respiratory Support 24 Hours After Transfer to ICU | | | |
| NC Oxygen > 4L/min | 4 (3.8) | 2 (5.7) | NS |
| HFNC | 33 (31.7) | 7 (20.0) | NS |
| CPAP | 2 (1.9) | 0 (0.0) | NS |
| BIPAP | 18 (17.3) | 4 (11.4) | NS |
| Mechanical Ventilation | 20 (19.2) | 2 (5.7) | NS |
| Does the Patient have a Tracheostomy? | | | |
| Yes | 16 (15.4) | 6 (17.1) | NS |
| If Tracheostomy, Specify Respiratory Support | | | |
| Humidified Air | 12 (11.5) | 6 (17.1) | NS |
| Humidified Oxygen | 2 (1.9) | 0 (0.0) | NS |
| Mechanical Ventilation | 2 (1.9) | 0 (0.0) | NS |
| Categories of Medications Administered in the 24 Hours Prior to Transfer to the ICU | | | NS |
| Inhaled Beta Agonists | 52 (50.0) | 7 (20.0) |  |
| Insulin | 0 (0.0) | 0 (0.0) |  |
| Anti-Seizure | 18 (17.3) | 4 (11.4) |  |
| Diuretics | 16 (15.4) | 1 (2.8) |  |
| Antihypertensives | 12 (11.5) | 2 (5.7) |  |
| Cardiac Medications | 15 (14.4) | 2 (5.7) |  |
| Anti-Sialagogues | 5 (4.8) | 0 (0.0) |  |
| IV Pressors/Inotropes | 0 (0.0) | 0 (0.0) |  |
| Opioids | 13 (12.5) | 11 (31.4) |  |
| ICU-Specific Medications Administered in the First 24 Hours of ICU Admission | | | NS |
| Pressors/Inotropes | 10 (9.6) | 0 (0.0) |  |
| Continuous Albuterol | 2 (1.9) | 1 (2.9) |  |
| Anti-hypertensives | 26 (25.0) | 9 (25.7) |  |
| Sedation/Analgesia | 0 (0.0) | 0 (0.0) |  |
| Endocrine/Metabolic Infusions | 3 (2.9) | 2 (5.7) |  |
| Did the Patient have an ICU Specific Procedure in the First 24 Hours | | | NS |
| Central Line | 0 (0.0) | 1 (2.9) |  |
| Arterial Line | 0 (0.0) | 1 (2.9) |  |
| Dialysis | 0 (0.0) | 0 (0.0) |  |
| Pericardiocentesis | 0 (0.0) | 0 (0.0) |  |
| ECMO | 0 (0.0) | 0 (0.0) |  |
| ICP Monitor/EVD | 0 (0.0) | 0 (0.0) |  |
| Cardiac Arrest 24 Hours Prior to Admission to the ICU | | | |
| Yes | 0 (0.0) | 0 (0.0) | NS |
| 1. Univariate comparisons were completed using the Barnard’s exact test. The Bonferroni correction was used to adjust p-values within each care factor division to control for Type 1 error.   Abbreviations: ICU = intensive care unit; q = every; h = hour; NC = nasal canula; HFNC = high flow nasal canula; CPAP = continuous positive airway pressure; BIPAP = bilevel positive airway pressure; ICP = intracranial pressure; EVD - external ventricular drain. | | | |
|  |  |  |  |
|  | **Prediction Outcome Groups** | | |
| **Transfer Group: ICU DISCHARGE**  **Care Factor Divisions** | **True Negative: Correct Predictions for Transfer out of the ICU (n = 50)** | **False Positive: Incorrect Predictions for no Transfer out of the ICU** | **adjusted p-value (1)** |
| Primary Diagnosis | | | |
| Respiratory Failure | 19 (38.0) | 36 (72.0) | **<0.01** |
| Neurologic Disorders | 7 (14.0) | 3 (6.0) | NS |
| Endocrine | 7 (14.0) | 1 (2.0) | NS |
| Trauma | 4 (8.0) | 0 (0.0) | NS |
| Ingestion | 3 (6.0) | 1 (2.0) | NS |
| Oncology | 3 (6.0) | 0 (0.0) | NS |
| Cardiac | 2 (4.0) | 2 (4.0) | NS |
| Hematology | 1 (2.0) | 0 (0.0) | NS |
| Renal | 1 (2.0) | 0 (0.0) | NS |
| Post-Operative Care | 1 (2.0) | 4 (8.0) | NS |
| Septic Shock | 1 (2.0) | 3 (6.0) | NS |
| Gastrointestinal | 1 (2.0) | 0 (0.0) | NS |
| Secondary Diagnosis | | | |
| Non-Septic Infection | 7 (14.0) | 7 (14.0) | NS |
| Post-Operative | 4 (8.0) | 0 (0.0) | NS |
| Hematology | 4 (8.0) | 0 (0.0) | NS |
| Neurologic | 3 (6.0 | 1 (2.0) | NS |
| Septic Shock | 2 (4.0) | 1 (2.0) | NS |
| Cardiac Disorders | 2 (4.0) | 3 (6.0) | NS |
| Solid Organ Transplant | 1 (2.0) | 0 (0.0) | NS |
| Gastrointestinal | 1 (2.0) | 1 (2.0) | NS |
| Respiratory Failure | 1 (2.0) | 3 (6.0) | NS |
| Oncology | 0 (0.0) | 1 (2.0) | NS |
| Tertiary Diagnosis | | | |
| Neurologic | 2 (4.0) | 0 (0.0) | NS |
| Gastrointestinal | 2 (4.0) | 0 (0.0) | NS |
| Hematology | 0 (0.0) | 2 (4.0) | NS |
| Post-Operative Care | 1 (2.0) | 1 (2.0) | NS |
| Respiratory Failure | 1 (2.0) | 1 (2.0) | NS |
| Oncology | 1 (2.0) | 0 (0.0) | NS |
| Solid Organ Transplant | 1 (2.0) | 0 (0.0) | NS |
| Septic Shock | 1 (2.0) | 0 (0.0) | NS |
| Renal | 1 (2.0) | 0 (0.0) | NS |
| Frequency of Vital Signs 24 Hours Prior to Transfer Out of the ICU | | | |
| q1h | 45 (90.0) | 43 (86.0) | NS |
| q4h | 1 (2.0) | 0 (0.0) | NS |
| Frequency of Vital Signs 24 Hours After Transfer Out of the ICU | | | |
| q3h | 1 (2.0) | 0 (0.0) | NS |
| q4h | 45 (90.0) | 43 (86.0) | NS |
| Frequency of Neurologic Assessments 24 Hours Prior to Transfer Out of the ICU | | | |
| q1h | 14 (28.0) | 3 (1.9) | **0.04** |
| q2h | 5 (28.0) | 6 (12.0) | NS |
| q3h | 3 (6.0) | 1 (2.0)) | NS |
| q4h | 19 (38.0) | 33 (66.0) | **0.02** |
| Frequency of Neurologic Assessments 24 Hours After Transfer Out of the ICU | | | |
| q3h | 3 (6.0) | 1 (2.0)) | NS |
| q4h | 14 (28.0) | 23 (46.0) | NS |
| Highest Level of Respiratory Support 24 Hours Prior to Transfer Out of the ICU | | | |
| NC Oxygen < 4L/min | 4 (8.0) | 9 (18.0) | NS |
| NC Oxygen > 4L/min | 2 (4.0) | 1 (2.0) | NS |
| HFNC | 14 (28.0) | 23 (46.0) | NS |
| BIPAP | 3 (6.0) | 9 (18.0) | NS |
| Mechanical Ventilation | 0 (0.0) | 1 (2.0) | NS |
| Highest Level of Respiratory Support 24 Hours After Transfer Out of ICU | | | |
| NC Oxygen < 4L/min | 16 (32.0) | 33 (66.0) | **<0.01** |
| NC Oxygen > 4L/min | 1 (2.0) | 0 (0.0 | NS |
| HFNC | 1 (2.0) | 3 (6.0) | NS |
| BIPAP | 0 (0.0) | 1 (2.0) | NS |
| Does the Patient have a Tracheostomy? |  |  |  |
| Yes | 0 (0.0) | 1 (2.0)) | NS |
| If yes, what level of respiratory support? | | | |
| Humidified Air | 0 (0.0) | 1 (2.0)) | NS |
| ICU-Specific Medications Administered 24 hours Prior to ICU Discharge |  | | NS |
| Pressors/Inotropes | 0 (0.0) | 0 (0.0) |  |
| Continuous Albuterol | 0 (0.0) | 1 (2.0) |  |
| Anti-hypertensives | 6 (12.0) | 4 (8.0) |  |
| Sedation/Analgesia | 10 (20.0) | 8 (16.0) |  |
| Endocrine/Metabolic Infusions | 7 (14.0) | 1 (2.0) |  |
| Categories of Medications Administered in the 24 Hours after Transfer Out of the ICU | | | NS |
| Inhaled Beta Agonists | 5 (8.0) | 17 (34.0) |  |
| Insulin | 7 (14.0) | 1 (2.0) |  |
| Anti-Seizure Medications | 3 (6.0) | 3 (6.0) |  |
| Diuretics | 7 (14.0) | 5 (10.0) |  |
| Antihypertensives | 2 (4.0) | 7 (14.0) |  |
| Cardiac Medications | 4 (8.0) | 3 (6.0) |  |
| Anti-Sialagogues | 1 (2.0) | 4 (8.0) |  |
| IV Pressors/Inotropes | 0 (0.0) | 1 (2.0) |  |
| ICU Specific Procedure 24 Hours Prior to Transfer Out of the ICU | | | NS |
| Central Venous Line | 1 (2.0) | 1 (2.0) |  |
| Arterial Line | 0 (0.0) | 0 (0.0) |  |
| Dialysis | 0 (0.0) | 0 (0.0) |  |
| Pericardiocentesis | 0 (0.0) | 0 (0.0) |  |
| ECMO | 0 (0.0) | 0 (0.0) |  |
| ICP Monitor/EVD | 0 (0.0) | 0 (0.0) |  |
| Cardiac Arrest 24 hours Prior to Discharge from the ICU? | | | |
| Yes | 0 (0.0) | 0 (0.0) | NS |
| Readmission to the ICU | | | |
| Yes | 0 (0.0) | 0 (0.0) | NS |
| 1. Univariate comparisons were completed using the Barnard’s exact test. The Bonferroni correction was used to adjust p-values within each care factor division to control for Type 1 error.   Abbreviations: ICU = intensive care unit; q = every; h = hour; NC = nasal canula; HFNC = high flow nasal canula; CPAP = continuous positive airway pressure; BIPAP = bilevel positive airway pressure; ICP = intracranial pressure; EVD – external ventricular drain. | | | |
|  |  |  |  |
|  | **Prediction Outcome Groups** | | |
| **Transfer Group: Non-transfer**  **Care Factor Divisions** | **True Negative: Correct Predictions for Patients Who Remain in Non-ICU care (n = 50)** | **False Positive: Incorrect Prediction of Transfer from Non-ICU to ICU (n = 50)** | **adjusted p-value (1)** |
| Primary Diagnosis | | | |
| Respiratory Failure | 17 (34.0) | 21 (42.0) | NS |
| Non-Septic Infection | 11 (22.0) | 9 (18.0) | NS |
| Gastrointestinal | 4 (8.0) | 4 (8.0) | NS |
| Hematology | 3 (6.0) | 1 (2.0) | NS |
| Neurologic Disorders | 4 (8.0) | 5 (10.0) | NS |
| Post-Operative Care | 4 (8.0) | 4 (8.0) | NS |
| Renal | 1 (2.0) | 1 (2.0) | NS |
| Cardiac Disorders | 1 (2.0) | 1 (2.0) | NS |
| Septic Shock | 1 (2.0) | 1 (2.0) | NS |
| Oncology | 1 (2.0) | 1 (2.0) | NS |
| Ingestion | 1 (2.0) | 1 (2.0) | NS |
| Endocrine | 1 (2.0) | 0 (0.0) | NS |
| Trauma | 1 (2.0) | 1 (2.0) | NS |
| Secondary Diagnosis | | | |
| Non-Septic Infection | 12 (24.0) | 12 (24.0) | NS |
| Gastrointestinal | 5 (10.0) | 6 (12.0) | NS |
| Respiratory Failure | 3 (6.0) | 2 (4.0) | NS |
| Post-Operative Care | 5 (10.0) | 4 (8.0) | NS |
| Neurologic Disorders | 2 (4.0) | 2 (4.0) | NS |
| Septic Shock | 1 (2.0) | 1 (2.0) | NS |
| Endocrine | 1 (2.0) | 1 (2.0) | NS |
| Cardiac Disorders | 1 (2.0) | 1 (2.0) | NS |
| Tertiary Diagnosis | | | |
| Non-Septic Infection | 6 (12.0) | 3 (6.0) | NS |
| Neurologic Disorders | 1 (2.0) | 2 (4.0) | NS |
| Gastrointestinal | 1 (2.0) | 0 (0.0) | NS |
| Post-Operative Care | 1 (2.0) | 0 (0.0) | NS |
| Respiratory Failure | 1 (2.0) | 1 (2.0) | NS |
| Cardiac Disorders | 1 (2.0) | 1 (2.0) | NS |
| Frequency of Vital Signs 24 Hours Prior to Time Period of Interest | | | |
| q1h | 26 (52.0) | 31 (62.0) | NS |
| q2h | 8 (16.0) | 6 (12.0) | NS |
| q3h | 6 (12.0) | 6 (12.0) | NS |
| q4h | 10 (20.0) | 7 (14.0) | NS |
| Frequency of Vital Signs 24 Hours After the Time Period of Interest | | | |
| q1h | 26 (52.0) | 31 (62.0) | NS |
| q2h | 8 (16.0) | 6 (12.0) | NS |
| q3h | 6 (12.0) | 6 (12.0) | NS |
| q4h | 10 (20.0) | 7 (14.0) | NS |
| Frequency of Neurologic Assessments 24 Hours Prior to Time Period of Interest | | | |
| q1h | 9 (18.0) | 7 (14.0) | NS |
| q2h | 2 (4.0) | 2 (4.0) | NS |
| q3h | 3 (6.0) | 3 (6.0) | NS |
| q4h | 9 (18.0) | 8 (16.0) | NS |
| q6h | 3 (6.0) | 3 (6.0) | NS |
| q8h | 1 (2.0) | 2 (4.0) | NS |
| q12h | 21 (42.0) | 21 (42.0) | NS |
| q24h | 2 (4.0) | 4 (8.0) | NS |
| Frequency of Neurologic Assessments 24 Hours After the Time Period of Interest | | | |
| q1h | 9 (18.0) | 7 (14.0) | NS |
| q2h | 2 (4.0) | 2 (4.0) | NS |
| q3h | 3 (6.0) | 3 (6.0) | NS |
| q4h | 9 (18.0) | 8 (16.0) | NS |
| q6h | 3 (6.0) | 3 (6.0) | NS |
| q8h | 1 (2.0) | 2 (4.0) | NS |
| q12h | 21 (42.0) | 21 (42.0) | NS |
| q24h | 2 (4.0) | 4 (8.0) | NS |
| Highest Level of Respiratory Support 24 Hours Prior to Time Period of Interest | | | |
| NC Oxygen < 4L/min | 9 (18.0) | 14 (28.0) | NS |
| NC Oxygen > 4L/min | 4 (8.0) | 4 (8.0) | NS |
| HFNC | 3 (6.0) | 2 (4.0) | NS |
| CPAP | 1 (2.0) | 1 (2.0) | NS |
| BIPAP | 1 (2.0) | 2 (4.0) | NS |
| Mechanical Ventilation | 2 (4.0) | 2 (4.0) | NS |
| Highest Level of Respiratory Support 24 Hours After the Time Period of Interest | | | |
| NC Oxygen < 4L/min | 9 (18.0) | 14 (28.0) | NS |
| NC Oxygen > 4L/min | 4 (8.0) | 4 (8.0) | NS |
| HFNC | 3 (6.0) | 2 (4.0) | NS |
| CPAP | 1 (2.0) | 1 (2.0) | NS |
| BIPAP | 1 (2.0) | 2 (4.0) | NS |
| Mechanical Ventilation | 2 (4.0) | 2 (4.0) | NS |
| Tracheostomy | | | |
| Yes | 2 (4.0) | 2 (4.0) | NS |
| If yes, what level of respiratory support? | | | |
| Humidified Air | 1 (2.0) | 1 (2.0) | NS |
| Categories of Medications were Administered in the 24 Hours Prior to Time Period of Interest | | | NS |
| Inhaled Beta Agonists | 14 (28.0) | 17 (34.0) |  |
| Insulin | 2 (4.0) | 1 (2.0) |  |
| Opioids | 8 (16.0) | 5 (10.0) |  |
| Anti-Seizure Medications | 8 (16.0 | 9 (18.0) |  |
| Anti-Sialagogues | 1 (2.0) | 1 (2.0) |  |
| Diuretics | 3 (6.0) | 3 (6.0) |  |
| Antihypertensives | 1 (2.0) | 1 (2.0) |  |
| Cardiac Medications | 0 (0.0) | 0 (0.0) |  |
| Categories of Medications were Administered in the 24 Hours after the Time Period of Interest | | | NS |
| Inhaled Beta Agonists | 14 (28.0) | 17 (34.0) |  |
| Insulin | 2 (4.0) | 1 (2.0) |  |
| Opioids | 8 (16.0) | 5 (10.0) |  |
| Anti-Seizure Medications | 8 (16.0 | 9 (18.0) |  |
| Anti-Sialagogues | 1 (2.0) | 1 (2.0) |  |
| Diuretics | 3 (6.0) | 3 (6.0) |  |
| Antihypertensives | 1 (2.0) | 1 (2.0) |  |
| Cardiac Medications | 0 (0.0) | 0 (0.0) |  |
| Did the Patient have a Procedure 24 Hours Prior to Time Period of Interest | | | NS |
| Central Venous Line | 1 (2.0) | 1 (2.0) |  |
| Arterial Line | 0 (0.0) | 0 (0.0) |  |
| Dialysis | 0 (0.0) | 0 (0.0) |  |
| Pericardiocentesis | 0 (0.0) | 0 (0.0) |  |
| ECMO | 0 (0.0) | 0 (0.0) |  |
| ICP Monitor/EVD | 0 (0.0) | 0 (0.0) |  |
| 1. Univariate comparisons were completed using the Barnard’s exact test. The Bonferroni correction was used to adjust p-values within each care factor division to control for Type 1 error.   Abbreviations: ICU = intensive care unit; q = every; h = hour; NC = nasal canula; HFNC = high flow nasal canula; CPAP = continuous positive airway pressure; BIPAP = bilevel positive airway pressure; ICP = intracranial pressure; EVD - external ventricular drain. | | | |
