# Supplementary material for: Clinical assessment of the criticality index – dynamic, a machine learning prediction model of future care needs in pediatric inpatients
Source: PLoS One. 2025 Apr 30;20(4):e0320586. doi: 10.1371/journal.pone.0320586 (PMC12043114; doi:10.1371/journal.pone.0320586)
Supplement: S3 Appendix — “True” is the correct prediction and “false” is the incorrect prediction. “Positive” is the ICU care location and “negative” is a non-ICU care location. (DOCX) [file pone.0320586.s003.docx]

Appendix 3: Reasons for ICU Admission, Discharge, or Non-ICU care. “True” is the correct prediction and “false” is the incorrect prediction. “Positive” is the ICU care location and “negative” is a non-ICU care location.

| **Reasons for ICU Admission^1^** | **True Positive**  (n = 104) | **False Negative**  (n = 35) |
| --- | --- | --- |
| **Respiratory Support** | n = 66 (63.5%) | n = 12 (34.3%) |
|  | - HFNC support - BiPAP with or without asthma - Need for continuous albuterol in a < 2 yo - Ventilator dependent at baseline - Need for mechanical ventilation | - HFNC support - BiPAP without asthma - Croup receiving racemic epinephrine more frequently than every 2 hours |
| **Neurologic Monitoring or Management** | n = 17 (16.3%) | n = 11 (31.4%) |
|  | - Neurologic monitoring - Seizure management - Increased ICP management/monitoring | - Neurologic monitoring - Concern for increased ICP - Post-operative craniotomy |
| **Cardiac Care** | n = 12 (11.5%) | n = 5 (14.3%) |
|  | - Post-operative care - Worsening cardiac output | - Arrhythmia management - New diagnosis of coronary anomaly |
| **Hemodynamic Instability** | n = 7 (6.7%) | n = 4 (11.4%) |
|  | - Septic shock - Concern for evolving septic shock requiring close monitoring | - Septic Shock - Hypovolemic shock - Hypothermia |
| **General Post-Operative Care** | n = 2 (1.9%) | n = 3 (8.6%) |
|  | - Post-operative care in a medically complex patient | - Post-operative care for ruptured appendicitis - Massive hemoptysis |
| **Reasons for ICU Discharge** | **True Negative**  (n = 50) | **False Positive**  (n = 50) |
| **Respiratory Support** | n = 19 (38.0%) | n = 38 (76%) |
|  | - Weaned to nasal cannula/room air - Weaned off BiPAP in the setting of status asthmaticus - Weaned to home respiratory settings of BiPAP at night | - Weaned to nasal cannula/room air - Weaned to a new baseline BiPAP settings at night |
| **Neurologic Monitoring or Management** | n = 13 (26%) | n = 4 (8.0%) |
|  | - Neurologic assessments spaced with improvement in mental status - Post-operative neurologic monitoring no longer required | - Neurologic assessments spaced with improvement in mental status - Post-operative neurologic monitoring no longer required - Resolution of concerns for ICP - Seizures resolved |
| **Diabetes Management** | n = 7 (14.0%) | n = 1 (2.0%) |
|  | - 1. DKA resolved | - 1. DKA resolved |
| **Hemodynamic Instability** | n = 4 (8.0%) | n = 3 (6.0%) |
|  | - Sepsis resolved - Hypertensive emergency resolved - Hypothermia resolved | - Sepsis resolved |
| **Hematology/Oncology** | n = 3 (6.0%) | n = 0 (0.0%) |
|  | - Transfusion for severe anemia completed - Tumor lysis syndrome resolved |  |
| **Ingestion Management** | n = 2 (4.0%) | n = 1 (2.0%) |
|  | - Acetomenophen ingestion resolved - Multi-substance ingestion resolved | - Aspirin ingestion resolved |
| **Cardiac Management** | n = 2 (4.0%) | n = 2 (4.0%) |
|  | - Arrhythmia management resolved with pacemaker placement | - Recovered from surgery - Arrhythmias resolved |
| **General Post-Operative Care** | n = 0 (0.0%) | n = 1 (2.0%) |
|  |  | - Adequately monitored post-operatively |
| **Reasons for inpatient care** | **True Negative**  (n = 50) | **False Positive**  (n = 50) |
| **Respiratory Support** | n = 20 (40.0%) | n = 22 (40%) |
|  | - Required low flow supplemental oxygen - Required albuterol every 2 hours or less frequently - Required breathing treatments other than albuterol every 2 hours or less frequently | - Required low flow supplemental oxygen - Continuous albuterol in a child > 3 - Weaned from HFNC in the ICU, stable on low flow oxygen - Weaned to a new baseline BiPAP settings at night (prior ICU care) - 2 NICU patients were coded as inpatient, but were found to actually be cared for in the NICU on CPAP |
| **General Post-Operative Care** | n = 8 (16.0%) | n = 7 (14.0%) |
|  | - Post-operative appendectomy management - Post-operative monitoring after an Intussusception reduction - Burn management - Monitoring following trauma | - Post-operative appendectomy management - Monitoring following trauma - Post-operative monitoring in an oncology patient who developed a post-operative infection |
| **Neurologic Monitoring or Management** | n = 4 (8.0%) | n = 5 (10.0%) |
|  | - Needed neurologic assessments every 2 hours or less frequently - Seizure management - Encephalitis management appropriate for management on inpatient unit | - Needed neurologic assessments every 2 hours or less frequently - Seizure management - Seizure management in a patient on chronic respiratory support - Encephalitis management appropriate for management on inpatient unit |
| **Gastro-intestinal Disorder Management** | n = 1 (2.0%) | n = 3 (6.0%) |
|  | - Required IVF | - Required IVF and medication management |
| **Cardiac Management** | n = 2 (4.0%) | n = 1 (2.0%) |
|  | - Hypertension management on oral medications only | - Hypertension management on oral medications only |
| **Hematology** | n = 2 (4.0%) | n = 2 (4.0%) |
|  | - Monitoring during transfusion in the setting of anemia | - Monitoring during transfusion in the setting of anemia |
| **Non-Septic Infection Management** | n = 10 (20.0%) | n = 8 (16.0%) |
|  | 1. Admitted for IV antibiotics 2. Neonatal infection observation | 1. Admitted for IV antibiotics 2. Neonatal infection observation 3. Admitted for IV antibiotics on baseline supplemental oxygen via tracheostomy |
| **Ingestion Management** | n = 1 (2.0%) | n = 1 (2.0%) |
|  | - Monitoring post ingestion | - Monitoring post ingestion |
| **Diabetes Management** | n = 2 (4.0%) | n = 1 (2.0%) |
|  | - Transitioned to subq insulin after admission for DKA | - Patient was eventually transferred to PICU for DKA in subsequent time periods |

1. All reasons for ICU admission, ICU discharge or inpatient care are listed in order of frequency

2. Abbreviations used: HFNC = high flow nasal cannula, BiPAP = =bi-level positive airway pressure, ICP = intracranial pressure, DKA = diabetic keto acidosis, subq = subcutaneous
